# Cerebral oxygenation during submaximal and peak exercise for sedentary adults with and without down syndrome

**DOI:** 10.3389/fphys.2025.1595710

**Published:** 2025-07-09

**Authors:** Pieter-Henk Boer

**Affiliations:** Cape Peninsula University of Technology, Cape Town, South Africa

**Keywords:** Prefrontal Cortex, brain oxygenation, cognition, NIRS, HHB, HbO2, VO2 peak

## Abstract

**Background:**

Individuals with Down syndrome (DS) are born with or develop many physical limitations which affect their quality of life. Although studies involving cerebral oxygenation in the general population have been performed, such a study is yet to be conducted on individuals with DS.

**Method:**

Fifty-four participants (DS:27; non-DS:27) were tested for cerebral oxygenation during an incremental exercise test. Participants (38.9 ± 5.9 years) were tested for ΔHbO_2_ and ΔHHb using near-infrared spectroscopy. V̇O_2_ peak determination was performed with a standardised incremental treadmill protocol and the ventilatory threshold (VT) and respiratory compensation point (RCP) were determined.

**Results:**

There were no significant differences for $\dot{V}$ O_2_ between 80%VT, VT, RCP and $\dot{V}$ O_2_ peak, as well as VT/ $\dot{V}$ O_2_ peak and RCP/ $\dot{V}$ O_2_ peak between DS and non-DS groups (p > 0.05). Significant differences between DS and non-DS were reported for ΔHbO_2_ in the left and right prefrontal cortex for both males and females (p < 0.05) with medium to large effect sizes, but not for ΔHHb (p > 0.05) (except for males regarding RPC). Significant main effects over exercise intensity for ΔHbO_2_ and ΔHHb were noted for both groups, genders and hemispheres, but differences with respect to the ΔHHb in transition from RCP to peak exercise.

**Conclusion:**

A significant different evolution for ΔHbO_2_ over exercise intensity (80% VT, VT, RCP, VO_2_ peak) between sedentary adults with and without DS was found. This ΔHbO_2_ may possibly reflect previously reported executive function limitations and/or the many physiological limitations that individuals with DS are born with or develop over time. Such speculations would need to be tested with future cause-effect studies.

## 1 Introduction

Down syndrome (DS) is a genetic condition affecting chromosomes, with a prevalence of 1 in 650 live births in developing countries (DSSA, 2023). Individuals with DS have a unique set of physical characteristics such as short stature, small low-set ears, flat nasal bridge, small mouth, and many other unique characteristics (Antonarakis et al., 2020). They have physical conditions that hamper their ability to perform submaximal and maximal exercise such as muscle hypotonicity, ligamentous laxity, congenital heart defects and chronotropic incompetence (Foley and Killeen, 2019; Zahari et al., 2019; Fernhall et al., 2013; Guerra et al., 2003). Chronotropic incompetence may be caused by an inadequate catecholamines response to maximal exercise in individuals with DS (Fernhall et al., 2009).

A variety of parameters associated with maximal exercise can be improved with structured aerobic or anaerobic, or combined training interventions consisting of walking, running, interval training, cycling, swimming, resistance training and many other modalities (Boer, 2020; Hardee and Fetters, 2017; Boer and Moss, 2016; Kerstiens and Green, 2015; Li et al., 2013; Mendonca et al., 2013; Shields et al., 2013; Mendonca et al., 2011; Mendonca and Pereira, 2009; Carmeli et al., 2002). However, some aerobic exercise intervention studies show limited improvement in peak oxygen consumption (Varela et al., 2001; Millar et al., 1993).

During exercise, the demand for oxygen by the body increases to meet the metabolic needs of the working muscles. The brain, like other organs, also requires oxygen to function properly (Ainslie et al., 2016; Hall et al., 2012). Maintaining adequate cerebral oxygenation during exercise is crucial for maintaining cognitive function and preventing neurological injury (Mekari et al., 2015; Raichle and Gusnard, 2002).

Several factors affect cerebral oxygenation during exercise, including the intensity, duration, and type of exercise, as well as the individual’s fitness level and health status (Oussaidene et al., 2015). During high-intensity exercise, there is a reduction in cerebral oxygenation due to increased demand for oxygen by the working muscles and decreased blood flow to the brain (Mekari et al., 2015; Drigny et al., 2014). This can result in symptoms such as dizziness, light-headedness, and in extreme cases loss of consciousness. However, regular exercise training can improve cerebral oxygenation by increasing blood flow to the brain and promoting the growth of new blood vessels (Coetsee and Terblanche, 2017). Additionally, certain types of exercise, such as aerobic exercise, have been shown to improve cognitive function and reduce the risk of neurological disorders such as dementia and Alzheimer’s disease which are conditions commonly affecting individuals with DS (Fortea et al., 2021). It is also known that individuals with DS have pronounced limitations in executive function (EF) as reported in a meta-analytic study (Tungate and Conners, 2021). However, EF and cognition may be improved with exercise as EF is associated with cerebral oxygenation (Goenarjo et al., 2020; Mekari et al., 2019; Ogoh and Tarumi, 2019; Ogoh, 2017; Dupuy et al., 2015). Although these studies were conducted in the general population, it has been demonstrated that cognition (Merzbach et al., 2023; Ptomey et al., 2018) and EF (Ringenbach et al., 2021) can be significantly improved for individuals with DS, which is important since most individuals with DS exhibit cognitive impairment (Moncaster et al., 2010).

Monitoring cerebral oxygenation during exercise can be done through non-invasive techniques such as near-infrared spectroscopy (NIRS), which measures changes in blood oxygenation levels in the brain. Typically, cerebral oxygenation is monitored during rest and a maximal aerobic incremental test and assessed at specific breakpoint intensities (such as the ventilatory threshold and the respiratory compensation point (RCP)) and at peak or maximum intensity (Kojima et al., 2021; Stevens et al., 2018; Tempest et al., 2017; Oussaidene et al., 2015; Tempest and Parfitt, 2016; Jung et al., 2015). In most cases the change in cerebral oxygenation increases from rest to the ventilatory threshold and RCP and either plateaus until peak exercise (Tempest et al., 2017; Tempest and Parfitt, 2016; Jung et al., 2015) or increases (Stevens et al., 2018; Tempest and Parfitt, 2016) or decreases (Kojima et al., 2021; Oussaidene et al., 2015). The discrepancy between RCP and peak exercise could be related to fitness (De Wachter et al., 2021).

Such a study remains to be conducted in individuals with DS and could provide further insight into the physical limitations experienced by these individuals during submaximal and peak exercise. Consequently, the aim with such an exploratory study was to assess the cerebral oxygenation and deoxygenation in sedentary adults with DS compared to an age-matched non-DS sedentary control group.

## 2 Methods

### 2.1 Participants

A total of 54 sedentary adults (DS: 27; 27 non-DS) with a mean age of 38.9 years (±5.9) were tested for body mass, stature, and cerebral oxygenation during a standardised incremental VO_2_ peak test. Testing was conducted within a 4-week period. Information about the study and the consent form were distributed to the Executive Managers of three Intellectually Disabled Care Centres (IDCC) who redistributed the information and consent forms to the parents/guardians of adults with DS. Information about the study was also distributed to adults in the general population within the same area(s) of the three IDCC via social media platforms and the local newspapers.

Before participating in the study, participants or the legal guardians (for adults with DS) completed a consent form, an adapted consent form with plain language (for adults with DS) and an adapted physical activity readiness questionnaire (aPARQ).

Individuals of 25–50 years of age were eligible to participate in the study. Furthermore, adults diagnosed with DS (for the DS group) who had the cognitive ability to perform and understand all testing instructions were eligible for the study. Individuals who had not taken part in any structural physical activity (structured aerobic [including walking], anaerobic or resistance training activity) during the 6 months prior to the study were included. Any individuals suffering from congenital heart disease or any other chronic physical or mental conditions contraindicative to the demand of physical activity, or those who did not answer ‘yes’ to all the questions in the aPARQ were excluded. The DS and non-DS groups were not purposely matched for exact physical activity profile or body mass index. This information was collected by the primary guardian of the participant (for the DS group). Adults who could not walk on the treadmill without holding onto the handrails were also excluded.

### 2.2 Procedures

Ethical approval for this study was granted by the Faculty of Health Sciences Research Ethical Committee from the University of Pretoria (269/2023).

The participants visited the laboratory on three occasions. Upon the first visit, the participants’ body mass and stature were assessed. Participants wore a shirt and light weight trunks only. The body mass and stature measurements were used to calculate body mass index (BMI). The participants were familiarised with the cerebral oxygenation and maximal oxygen uptake equipment (first and second visit) as stipulated by Fernhall et al. (1990). They practiced walking on the treadmill using the standardised protocol adapted for adults with and without DS using gas exchange equipment (Boer, 2023; Fernhall et al., 1990). Upon the third visit, all participants performed the peak oxygen uptake test in association with cerebral oxygenation. More familiarisation sessions were conducted if deemed necessary.

All participants were tested using the Cosmed K5 CPET testing system (Cosmed, Rome, Italy) using a standardised treadmill protocol. The treadmill protocol started with an initial velocity of 4 km/h at an incline of 0%. After two-minute intervals, the treadmill incline increased by 2.5% until it reached an incline of 15%, whereafter the speed increased by 1.6 km/h after every minute until exhaustion was reached (Boer, 2023; Fernhall et al., 1990). The treadmill was calibrated, and the running belt moved freely with no obstructions. The $\dot{V}$ O_2_ peak measurement was taken as the single highest recorded measurement during the final 30 s of the test, after the data was filtered to 10-second periods. The participants were continuously encouraged to continue until maximal exhaustion was reached (Varela et al., 2001). Two exercise physiologists visually determined the ventilatory threshold (VT) and respiratory compensation point (RCP) (Marillier et al., 2022; Wasserman, 1984). The VT was determined as an increase in $\dot{V}$ E/ $\dot{V}$ O_2_ and PETO_2_ and no increase in $\dot{V}$ E/ $\dot{V}$ CO_2_. The RCP was determined where $\dot{V}$ E, $\dot{V}$ E/ $\dot{V}$ O_2_, $\dot{V}$ E/ $\dot{V}$ CO_2_, PETCO_2_ concomitantly increased, but PETCO_2_ decreased during the incremental exercise test. If discrepancies arose between the two exercise physiologists regarding the specific threshold point, a third exercise physiologist were approached for final determination. To determine whether a valid $\dot{V}$ O_2_ peak test was achieved, absolute $\dot{V}$ O_2_ values of less than 150 mL/min increase during the final 60 s of exercise, despite increasing workload were assessed for each participant. According to the American College of Sports Medicine, the plateau in $\dot{V}$ O_2_ is considered a gold standard indicator that an individual has fully exerted their aerobic capacity (Ozemek et al., 2025). Immediately after exercise cessation, heart rate recovery (HRR) was calculated during the first and second minutes of recovery.

Changes in oxyhaemoglobin (HbO_2_) and deoxyhaemoglobin (HHb) was measured by a two-channel near-infrared spectrometer (NIRO 200NX, Hamamatsu, Japan) at rest and during completion of the peak oxygen uptake test. Prior to any measurements being taken, the participant’s forehead was cleaned with alcohol swabs to avoid false measurements caused by make-up, or other potential substances on the surface of the skin. Measurements were made with the sampling time set at 5 Hz (Hz) and at wavelengths of 735, 810, and 850 nm as determined by the manufacturer. The position of the measurement probes was calculated using the international 10–20 system for EEG electrode placement (Jurcak et al., 2007). The light emitter sensor was placed on the medial side of the forehead for the prefrontal cortex measurements, at Fp1 and Fp2 for the left- and right-hand side, respectively, with the detectors being placed between positions F3 and F7 on the left-hand side, and F4 and F8 on the right-hand side. The distance between each emitter and detector was 4 cm.

Before the exercise test was performed, participants were instructed to sit quietly for 5 min with their eyes closed while resting values were obtained. Seeing that it is not possible to assess optical path lengths with NIRS, relative changes in HbO_2_ and HHb with respect to baseline were analysed. The concentration of HbO_2_ represents the balance between oxygen distribution and oxygen use; HHb reflects oxygen extraction by the tissue (Coetsee and Terblanche, 2017). Furthermore, a white Velcro headband was placed around the head to ensure that the wires of the two measurement channels did not impede the participant in any way. Markers were placed and the time at the beginning and end of each event (end of rest or increase in treadmill incline or speed in maximal incremental test) in the testing procedure was recorded to ensure accuracy of start and end times. All cerebral hemodynamic recordings were averaged during the last 30 s of a 5-minute seated resting period and during the final 30 s of each incremental exercise stage including up to the point of peak exercise.

### 2.3 Statistical analysis

Data were analysed with the Statistical Package for the Social Sciences (SPSS 30.0, Chicago, IL, United States). A significance level of p < 0.05 was used. A sample size of 10 or more afforded adequate statistical power for between-group differences, using an alpha of 0.05 and a power of 0.80 from standard deviations in other studies where untrained individuals were tested (Stevens et al., 2018; Tempest and Parfitt, 2016). All data were tested for normality and homogeneity of variance with the Shapiro-Wilk statistic and the Levene’s test respectively. Data are presented as mean and standard deviation. Differences between groups at baseline, were evaluated using an independent t-Test for demographic, anthropometrical, submaximal and peak oxygen uptake.

A two-way ANOVA with repeated measures (80% VT, VT, RCP and End) and between groups (DS vs. non-DS) was used to identify the interaction effect of exercise intensity and group on ΔHbO_2_ and ΔHHb separately as conducted by Stevens et al. (2018) and Brugniaux et al. (2014). Consequently, the analysis involved eight separate ANOVA models, stratified by sex (male and female), haemoglobin species (HbO_2_ and HHb), and NIRS probe location (left and right prefrontal cortex). Greenhouse-Geisser corrections were applied if the assumption of sphericity was not met. Furthermore, a two-way ANOVA with repeated measures (80% VT, VT, RCP and End) and between groups (DS vs. non-DS) was used to identify the interaction effect of P_ET_CO_2_ for males and females separately. When statistical significance was detected through the two-way ANOVA, all pairwise differences were identified using Bonferroni adjusted pairwise comparisons. Effect sizes associated with the F statistics were expressed as partial eta-squared (n_p_^2^) defined as small (0.01), medium (0.06) and large (0.14) (Cohen et al., 1990).

## 3 Results

All 54 participants completed the $\dot{V}$ O_2_ peak test without any problems or injuries. Participant demographics, anthropometry, heart rate, heart rate recovery, submaximal and peak $\dot{V}$ O_2_ values are presented in Table 1 for the two groups and categorised according to gender. No significant differences between groups for each gender were found at baseline, except for height, body mass and peak HR for both males and females. Despite increasing workloads for all participants, a plateau in peak $\dot{V}$ O_2_ values of a less than 150 mL/min increase in $\dot{V}$ O_2_ values presented during the final 60 s of exercise. There were no missing values for the metabolic data but there were two missing values each for NIRS data for males (LPC) and females (RPC) due to signal interference or noise.

**TABLE 1:** Participant demographics and average 30-s oxygen uptake ( $\dot{V}$ O_2_) for the Down syndrome and general population groups, reported as means (and standard deviations).

	Down syndrome (n = 27)		General population (n = 27)	
	Male (n = 16)	Female (n = 11)	Male (n = 15)	Female (n = 12)
Demographics				
Age (yrs)	38.4 (7.5)	38.0 (4.0)	39.1 (6.4)	40.1 (4.5)
Height (cm)	160.4 (8.3)	147.3 (5.0)	182.3 (10.1)*	165.2 (6.7)*
Body mass (kg)	75.0 (23.2)	65.1 (9.7)	102.1 (20.2)*	81.8 (16.4)*
BMI (kg/m_2_)	28.9 (7.8)	29.9 (3.6)	31.0 (7.0)	30.2 (7.1)
$\dot{V}$ O_2_ (mL/kg/min)				
80% of VT	19.1 (3.7)	18.4 (1.9)	18.9 (4.0)	18.0 (4.1)
VT	23.9 (4.7)	23.0 (2.3)	23.7 (5.0)	22.5 (5.2)
RCP	27.0 (5.9)	25.2 (3.3)	29.0 (7.7)	26.0 (6.6)
End (V̇O_2_ peak)	31.5 (7.1)	29.1 (4.3)	34.6 (9.0)	29.1 (7.2)
% of $\dot{V}$ O_2_ peak				
VT	76.9 (8.0)	79.8 (7.4)	70.7 (13.3)	78.2 (8.0)
RCP	86.2 (5.5)	86.7 3.5)	84.2 (7.2)	89.5 (4.0)
Heart rate (bpm)				
HR Peak	151.1 (28.3)	164.2 (17.3)	181.6 (14.7)*	180.0 (9.9)*
HRR-1 minute	25.6 (7.2)	31.4 (9.9)	28.0 (6.7)	23.5 (8.9)
HRR-2 minutes	41.1 (15.4)	46.6 (10.9)	47.1 (8.3)	45.4 (13.1)

*p < 0.01: significant difference between DS, and non-DS; BMI: body mass index; HR: heart rate; RCP: respiratory compensation point; VT: ventilatory threshold.

To determine whether any significant difference in submaximal and peak $\dot{V}$ O_2_ existed (as indicated by $\dot{V}$ O_2_) between the groups, at time points corresponding to different intensities of exercise (80% of VT, VT, RCP, peak exercise, VT/ $\dot{V}$ O_2_ peak and RCP/ $\dot{V}$ O_2_ peak), a group (2) by exercise intensity (4) mixed model ANOVA was conducted. The analysis showed no significant difference between groups for both males and females (p > 0.05). As expected, the main effect over time for $\dot{V}$ O_2_ was significantly increased from VT to RCP to $\dot{V}$ O_2_ peak for each group and gender (p < 0.001).

### 3.1 Change in HbO_2_

Regarding the males, there was a significant interaction effect for group (DS vs. non-DS) and time (over exercise intensity) (F (2.558; 69.079) = 4.528; p = 0.009; n_p_^2^ = 0.144) for the left prefrontal cortex (LPC) and the right prefrontal cortex (RPC) (F (2.123; 61.563) = 3.238; p = 0.026; n_p_^2^ = 0.104). Similarly, there was a significant interaction effect for group and time (F (1.6; 33.604) = 5.636; p = 0.012; n_p_^2^ = 0.212) for the LPC and the RPC (F (1.558; 29.593) = 6.305; p = 0.009; n_p_^2^ = 0.249) for the females. All effect sizes were large (medium for males for RPC) for the interaction model.

**FIGURE 1:**
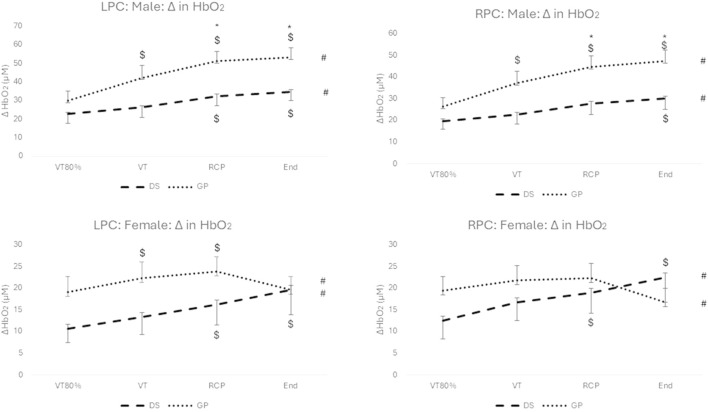
Change in HbO_2_ for males (top) and females (bottom), and for the LPC (left) and RPC (right) for the DS group (dashed line) and the non-DS group (dotted line). #: p < 0.05 for within group main effect of exercise intensity; *: p < 0.05 between DS and non-DS; $: p < 0.05 for exercise intensity versus VT80%.

Significant increases over exercise intensity (see # in Figure 1) with large effects sizes were reported for the males (LPC: F (2.558; 69.079) = 39.525, p < 0.001; n_p_^2^ = 0.594; RPC: F (2.123; 61.563) = 26.762; p < 0.001; n_p_^2^ = 0.489) and for the females (LPC: F (1.6; 33.604) = 6.917; p = 0.005; n_p_^2^ = 0.248; RPC: F (1.558; 29.593) = 5.253; p = 0.044; n_p_^2^ = 0.146). The *post hoc* Bonferroni analysis reported no significant differences (p > 0.05) from RCP to peak exercise for both hemispheres and both genders for DS and non-DS group (ΔHbO_2_ continued to increase for all males and for females with DS; but not for females in the non-DS group). Significant *post hoc* differences between DS and non-DS for RCP and peak exercise are reported for the males only (LPC and RPC) (See * in Figure 1). Significant within group *post hoc* increases are reported for exercise intensity versus VT80% (See $ in Figure 1).

### 3.2 Change in HHb

No significant interaction effect for group (DS vs. non-DS) and time (over exercise intensity) (F (1.886; 50.929) = 2.897; p = 0.067; np2 = 0.097) for the LPC was found for the males. Likewise, there was no significant interaction effect for group and time (F (1.465; 30.763) = 1.482; p = 0.241; n_p_^2^ = 0.066) for the LPC and the RPC (F (1.434; 27.242) = 0.700; p = 0.460; n_p_^2^ = 0.036) for the females. However, for the males a marginal significant interaction effect with a medium effect size (F (1.559; 45.208) = 3.659; p = 0.044; n_p_^2^ = 0.112) for the RPC was found.

**FIGURE 2:**
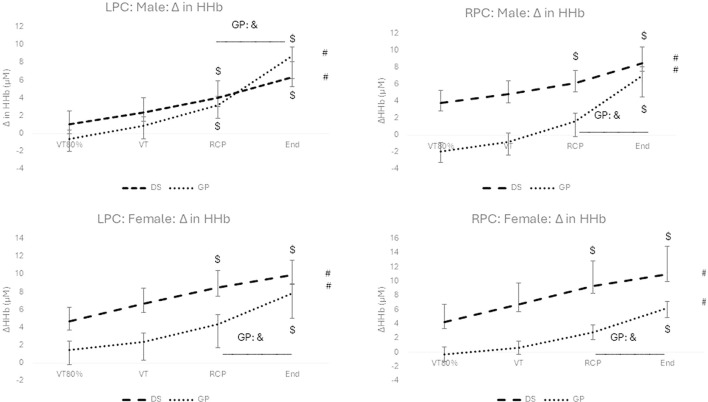
Change in HHb for males (top) and females (bottom), and for the LPC (left) and RPC (right) for the DS group (dashed line) and the non-DS group (dotted line). #: p < 0.05 for within group main effect of exercise intensity; *: p < 0.05 between DS and non-DS; $: p < 0.05 for exercise intensity versus VT80%; &:p < 0.05 significant increase from RCP to peak exercise.

Significant increases over exercise intensity (see # in Figure 2) with large effects sizes were reported for the males (LPC: F (1.886; 50.929) = 31.966; p < 0.001; n_p_^2^ = 0.542; RPC: F (1.559; 45.208) = 33.949; p < 0.001; n_p_^2^ = 0.538) and for the females (LPC: F (1.465; 30.763) = 33.399; p < 0.001; n_p_^2^ = 0.614; RPC: F (1.434; 27.242) = 26.090; p < 0.001; n_p_^2^ = 0.579). Although, ΔHHb continued to increase from RCP to peak exercise for both groups, both genders and both hemispheres, only the change for the non-DS group was significant (p < 0.05) (See and in Figure 2). Significant *post hoc* differences between DS and non-DS for RCP and peak exercise are reported for the males only (LPC and RPC) (See * in Figure 2). Significant within group *post hoc* increases are reported for exercise intensity versus VT80% (See $ in Figure 2).

### 3.3 Change in P_ET_CO_2_

**FIGURE 3:**
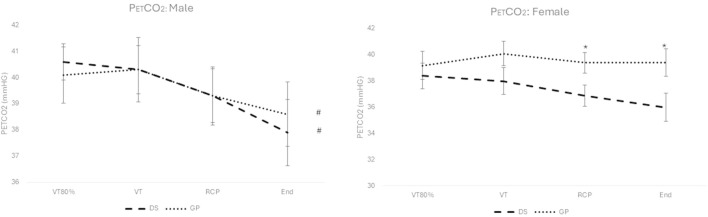
P_ET_CO_2_ for males (left) and females (right), for the DS group (dashed line) and the non-DS group (dotted line). #: p < 0.05 for within group main effect of exercise intensity; *: p < 0.05 between DS and non-DS.

No significant interaction effect (Figure 3) for group (DS vs. non-DS) and time (over exercise intensity) was reported for the males (F (1.779; 52.181) = 0.356; p = 0.680) and females (F (1.513; 31.775) = 1.389; p = 0.260). The only significant *post hoc* differences (p < 0.05) between DS and non-DS are reported for the females at the exercise intensity of the RCP and peak exercise (See * in Figure 3).

A significant decrease for the main effect over exercise intensity (see # in Figure 3) is reported for the males (F (1.799; 52.181) = 7.186; p = 0.002), but not for the females (F (1.513; 31.775) = 1.989; p = 0.162).

## 4 Discussion

The primary purpose of this exploratory study was to assess the change in HbO_2_ and HHb between sedentary adults with and without DS during an incremental VO_2_ peak test. A shared cerebral haemodynamic response between both groups of participants in the current study was that an increased change in the main effect of exercise intensity for the change in HbO_2_ and HHb for both hemispheres were reported as other studies conducted in the general population for sedentary and active individuals (Kojima et al., 2021; Stevens et al., 2018; Tempest et al., 2017; Oussaidene et al., 2015; Tempest and Parfitt, 2016; Jung et al., 2015) and confirmed by a systematic review (De Wachter et al., 2021). Similarly both male groups demonstrated a significant decrease over exercise intensity for P_ET_CO_2_ (main effect over time) but not for either group of females.

### 4.1 Profile of the change in HbO_2_ and HHb between individuals with DS and non-DS

Although there were no significant differences between DS and non-DS in submaximal (80% VT, VT and RCP) and $\dot{V}$ O_2_ peak values and VT and RCP expressed as a percentage of $\dot{V}$ O_2_ peak (males and females) and no significant interaction effect over exercise intensity for P_ET_CO_2_ (males and females) the current study reported a significant different evolution between groups (p < 0.05) for ΔHbO_2_ in the LPC and the RPC for males and females with medium to large effect sizes. On the other hand, no significant interaction effect was reported for ΔHHb, except for males regarding the RPC (p < 0.05). The fact that the interaction effect for ΔHHb was only significant for males for the RPC (p = 0.04) and not for the LPC (p = 0.07), is demonstrated by a significant increase in the change of HHb from RCP to peak exercise for non-DS men. It is difficult to attribute reasons for this finding, as the interaction effect is marginal for the RPC and not significant for the LPC or the female group. Future studies with larger sample sizes and the inclusion of possible molecular mechanisms could provide more distinct information on these initial exploratory findings and whether a possible gender or hemisphere difference exist for individuals with DS, and for what reason (Stone et al., 2016).

#### 4.1.1 Possible speculative mechanisms associated with the altered profiles for ΔHbO_2_ and ΔHHb

The mechanisms for these findings remain unexplained as functional magnetic resonance imaging (fMRI), positron emission topography and the assessment of other contributing molecular and physiological mechanisms such as insulin-like growth factor, vascular endothelial growth factor, vascular availability of nitric oxide, brain derived neurotrophic factor, neural connectivity and mitochondrial density were not studied (Brugniaux et al., 2014; Hayes et al., 2013). A large percentage of adults with DS develop Alzheimer’s disease (Zammit et al., 2024; Rubenstein et al., 2024; Fortea et al., 2021) and although none of the participants in the current study were diagnosed as having Alzheimer’s, it could be speculated that some of these sedentary and mostly overweight participants with a mean age of 38.2 years (56% older than 40 years) could have potentially developed some form of neurological degeneration, cerebral amyloid angiopathy, haemorrhagic lesions, white matter microstructural abnormalities or enlarged perivascular spaces (Rizvi et al., 2022; Fortea et al., 2021; Hasina et al., 2022). Amyloid plaques and tau neurofibrillary tangles are present in almost all individuals with DS by age 40 years (Zammit et al., 2024; Helman et al., 2019; Mann, 1988). There is evidence that the accumulation of amyloid plaque may cause damage to cerebral vasculature and brain cells in persons with Alzheimer’s disease (Lim et al., 2014; Jack et al., 2009; Buckner et al., 2005). However, these cause-effect speculations would need to be confirmed in an exclusive DS population.

A multitude of other factors could also be hypothesized to be related to this ΔHbO_2_ group difference seeing that most adults with DS suffer from chronotropic incompetence (Leti et al., 2015), muscle hypotonicity and ligamentous laxity (Mendonca et al., 2010), obesity and related conditions (Bertapelli et al., 2016), mitochondrial dysfunction (Tan et al., 2023), congenital heart disease (Santoro and Steffensen, 2021), thyroid disease (Capone et al., 2018), and blunted catecholamine response during submaximal and maximal exercise (Fernhall et al., 2009). The significantly ΔHbO_2_ profile could possibly be associated with the pronounced EF limitations for adults with DS as reported by a meta-analysis of 57 studies comparing DS to a typically developed mental age-matched group (Tungate and Conners, 2021). The prefrontal cortex is involved where most of the EFs occur (Bishop et al., 2010). Furthermore, cerebral oxygenation is important in influencing cognitive function (Ogoh and Tarumi, 2019; Ogoh, 2017) and several studies report the impact of cerebral oxygenation on EF during exercise and resting conditions (Goenarjo et al., 2020; Mekari et al., 2019; Dupuy et al., 2015).

#### 4.1.2 Fitness, physical activity, exercise and cerebral oxygenation

Although the mechanisms for these group differences are not known, exercise or being physically active would be important for both sedentary groups of the current investigation. It has been reported that exercise improves the density and integrity of grey and white matter (d’Arbeloff, 2020; Kundu et al., 2021) and other known cerebrovascular benefits such as an improvement in angiogenesis, neurovascular plasticity and oxygen saturation in brain regions related to cognitive performance such as the prefrontal cortices and hippocampus (Song et al., 2024; Stimpson et al., 2018). As reported by numerous other studies on the general population, the cerebral oxygenation profile improved after an exercise intervention (Marillier et al., 2022; Caen et al., 2019; Oussaidene et al., 2015) and this was confirmed by a recent systematic review (Salzman et al., 2022). This finding has also been shown in a cross-sectional study where a physical active group was compared to a sedentary group (Brugniaux et al., 2014). Future studies need to be conducted to confirm whether the cerebral oxygenation status could improve prefrontal cortex oxygenation for adults with DS with exercise or for those who are more physically active. If so, such interventions should be conducted as most adults with DS live a sedentary lifestyle as reported by a systematic review of 17 studies (Agiovlasitis et al., 2020) and age prematurely (Boer, 2024). Although this population ages prematurely, individuals with DS older than 40 years are a rapidly growing population due to improvements in medical care and the management of co-occurring illnesses, although these medical interventions do not help to improve the quality of life in older age (de Graaf et al., 2017). Fortunately, the positive effect of exercise for this population has been shown for health (Kerstiens and Green, 2015), fitness (Montalva-Valenzuela et al., 2024), functional ability and quality of life (Hardee and Fetters, 2017), cognition (Merzbach et al., 2023; Ptomey et al., 2018) and executive function (Ringenbach et al., 2021).

### 4.2 Change in HbO_2_ and HHb from low intensity exercise to peak exercise intensity

From low to submaximal exercise intensities, the change in cerebral oxygenation and deoxygenation is expected to increase (Jung et al., 2015). A significant main effect increase for ΔHbO_2_ and ΔHHb over exercise intensity was shown for both genders and groups with large effect sizes for both the LPC and RPC (p < 0.05). Most studies with trained and untrained participants have reported increased cerebral oxygenation from rest to VT and VT to RCP, where some showed a continued improvement to VO_2_ peak (Stevens et al., 2018; Tempest and Parfitt, 2016), or a plateau (Tempest et al., 2017; Tempest and Parfitt, 2016; Jung et al., 2015) or a decrease (Kojima et al., 2021; Oussaidene et al., 2015). Our study showed increases in the ΔHbO_2_ from 80% VT to VT and VT to RCP, but differences with respect to gender and group from RCP to peak VO_2_. However, ΔHbO_2_ ameliorations from RCP to peak exercise were not significant for both genders and for both the LPC and RPC (p > 0.05). In studies comparing trained versus untrained individuals a decreased cerebral oxygenation profile from RCP to peak exercise is reported for trained individuals (Oussaidene et al., 2015; Brugniaux et al., 2014) but only Oussaidene et al. (2015) reported significance. It is possible that for untrained individuals, this effect may not be reached due to a sedentary lifestyle (Stevens et al., 2018; Jung et al., 2015; Tempest and Parfitt, 2016). To study the discrepancies between studies, the direct analysis of cerebral blood flow and cerebral blood volume and other molecular mechanisms would need to be analysed at exercise intensities ranging from RCP to peak exercise as cerebral blood flow would decrease if metabolic acidosis was reached due to the increased ventilatory response resulting in a response to reduced PaCO_2_, known as hypocapnia (Caen et al., 2019). The current study did report a main effect of significant decrease over P_ET_CO_2_ for both groups of male participants over exercise intensity (p < 0.01) but not between RCP and peak exercise. There was no significant decrease over exercise intensity nor specifically between RCP and peak exercise for both groups of female participants for P_ET_CO_2_ although *post hoc* analyses reported significant differences between DS and non-DS females at RCP and peak exercise (Figure 3). What is apparent from these complex findings is that the observed differences in the ΔHbO_2_ cannot simply be ascribed to differences in the P_ET_CO_2_ kinetic given that both the hyper and hypocapneic profile responses to exercise were comparable across groups (especially for the males).

Regarding the ΔHHb, other studies conducted on trained and untrained individuals in the general population have also reported increases in the ΔHHb from rest to VT, VT to RCP and RCP to peak exercise (Kojima et al., 2021; Stevens et al., 2018; Tempest and Parfitt, 2016; Oussaidene et al., 2015). Although these studies demonstrated increases, they did not always report on statistical significance. The current study also reported increases in the change of HHb from 80%VT to VT, VT to RCP and from RCP to peak exercise for both genders and for the LPC and the RPC. Considering increases from RCP to peak exercise in both the LPC and the RPC, significance was reported for both genders for the non-DS group (p < 0.05) but not for the DS group. Mixed results are reported regarding this finding for other studies conducted on untrained individuals in the general population. One study reported significant increases (Tempest et al., 2017), some reported no significant increases (Caen et al., 2019; Oussaidene et al., 2015) and some did not report on significance (Stevens et al., 2018). We cannot attribute physiological and molecular mechanisms for these findings, and this is made more difficult due to discrepancies in other untrained general population studies, and the possible influencing factors regarding the many physiological and developed limitations faced by the DS group and their pronounced limitations in EF and cognition. Future studies need to be conducted to further analyse these findings, and the use of fMRI and positron emission topography could provide evidence of changes in the direct measurement of cerebral blood volume and cerebral blood flow.

### 4.3 Conclusion, limitations and future studies

The conclusion of the current study is that a significant different evolution of the ΔHbO_2_ existed for this group of sedentary adults with DS when conducting an incremental exercise test, although no significant between group differences with respect to VT, RCP, peak exercise and VT/ $\dot{V}$ O2 peak and RCP/ $\dot{V}$ O2 peak were noted. We cannot elaborate on the mechanisms of these findings, as further studies would be required. A limitation of the current study is the small sample size used for the female groups, although statistical sample size calculations reported adequate numbers. Caution is warranted to generalise these findings, but they do provide initial exploratory evidence of the ΔHbO_2_ and ΔHHb during a standardised incremental exercise test between adults with and without DS. Although all participants in the current study were sedentary and not involved in any type of structured physical activity for the 6 months prior to the study, the physicality of their jobs and work environment (for example, number of steps per day) may have differed, but this could be true for both groups. In future studies physical activity should be assessed using appropriate instruments to assess exact activity and spontaneous physical activity such as activity diaries or questionnaires (Agiovlasitis et al., 2020). Another limitation of the current study is that prefrontal cortical oxygenation measured via NIRS may be overestimated due to increased blood flow in the skin on the forehead (Miyazawa et al., 2013). However, the influence of skin blood flow was minimised as other studies by the correction of light penetration depth and the use of reference measurement locations as discussed in the methods section. Lastly, data regarding other potential co-morbidities or use of medications that could possibly affect cerebral oxygenation were not collected. Future studies should explore the possible mechanisms associated with the findings of the current study and whether the physiological and developed limitations experienced by sedentary adults with DS are related to cerebral oxygenation. Furthermore, studies using exercise interventions should provide evidence whether the cerebral oxygenation and deoxygenation profile could be altered and how this could be related to cognition using executive or cognitive tasks during rest and exercise for individuals with DS.

## Data Availability

The raw data supporting the conclusions of this article will be made available by the authors, without undue reservation.
